# Oligometastatic Bladder Cancer: Current Definitions, Diagnostic Challenges, and Evolving Therapeutic Strategies

**DOI:** 10.3390/cancers18020189

**Published:** 2026-01-07

**Authors:** Kieran Sandhu, David T. Hopkins, Matilda Newton, Niranjan Sathianathen, Sachin Perera, Nathan Lawrentschuk, Declan Murphy, Marlon Perera

**Affiliations:** 1 Division of Cancer Surgery, Peter MacCallum Cancer Centre, Melbourne 3000, Australia; 2 University of Cambridge, Cambridge CB2 1SZ, UK; 3 Department of Urology, Tallaght University Hospital, D24 NR0A Dublin, Ireland; 4 Department of Pharmacy, Royal Melbourne Hospital, Melbourne 3050, Australia; 5 Department of Pharmacy, Canberra Health Services, Canberra 2601, Australia; 6 Department of Surgery, University of Melbourne, Austin Health, Melbourne 3010, Australia; 7 Department of Urology, Royal Melbourne Hospital, Melbourne 3050, Australia; 8 Department of Surgery, University of Melbourne, Royal Melbourne Hospital, Melbourne 3050, Australia; 9 EJ Whitten Prostate Cancer Research Centre, Epworth Healthcare, Melbourne 3002, Australia; 10 Sir Peter MacCallum Department of Oncology, University of Melbourne, Melbourne 3010, Australia

**Keywords:** bladder cancer, metastases directed therapy, oligometastatic disease, oligometastatic bladder cancer

## Abstract

*Background*: Oligometastatic bladder cancer (OMBC) is increasingly recognised as an intermediate state between localised and widespread metastatic disease, although its definition and optimal management remain uncertain. Patients with OMBC have a generally more favourable prognosis compared to patients with metastatic disease. However, its definition, diagnostic criteria, and optimal management remain poorly standardised. *Methods*: This narrative review summarises current evidence on the definitions, diagnostic approaches, and treatment strategies for OMBC, with an emphasis on emerging biological and molecular insights that may refine disease classification and guide therapy. *Results*: Existing definitions of OMBC rely on lesion count and anatomical distribution, overlooking molecular and clinicopathological heterogeneity that influences prognosis and treatment response. Advances in Positron Emission Tomography (PET)/Computed Tomography (CT) and magnetic resonance imaging (MRI) have improved detection of small-volume disease, while liquid biopsy and circulating tumour DNA show promise for assessing micrometastatic burden. Therapeutic approaches, including metastasis-directed and consolidative therapies, are under investigation. Nonetheless, most data are derived from small, retrospective series, and evidence from prospective studies remains limited. *Conclusions*: Prospective, biomarker-integrated, and randomised trials are essential to refine definitions, optimise patient selection for therapy, and define the role of precision-based multimodal therapy in OMBC management.

## Simple Summary

Oligometastatic bladder cancer (OMBC) is increasingly described as an intermediate state between localised and widespread metastatic disease, although its definition and optimal management remains uncertain. This narrative review summarises current evidence relating to the definition, diagnosis, and treatment of OMBC, with an emphasis on recent advances that may refine patient selection and decision-making. We discuss existing anatomical definitions based on metastatic burden and distribution, alongside emerging data highlighting the importance of tumour biology, molecular alterations, and treatment responsiveness. Recent advances in imaging modalities, including PET/CT and MRI, are discussed, and we explore the role of circulating biomarkers such as tumour DNA for the detection of micrometastatic disease. Furthermore, we discuss therapeutic strategies including systemic therapy, metastasis-directed interventions, and consolidative approaches. Ultimately, future biomarker-driven prospective clinical trials are necessary to optimise treatment sequencing and clarify the role of precision-based multimodal therapy in OMBC.

## 1. Introduction

Bladder cancer (BC) is the ninth most common cancer worldwide, with its incidence steadily rising worldwide [1]. Most patients are diagnosed early, but of the 5% presenting with metastatic disease, five-year overall survival (OS) rates remain under 5% [2]. Historically, platinum-based chemotherapy achieved median OS rates of approximately 14–19 months [3,4]. However, the recent EV-302/KEYNOTE-A39 trial has demonstrated superior outcomes with Enfortumab Vedotin plus Pembrolizumab, which is now considered the standard first-line regimen for locally advanced or metastatic urothelial carcinoma, achieving a median OS of 33.8 months (95% CI: 26.1–39.3 months) compared to 15.9 months (95%CI: 13.6–18.3 months) for platinum-based therapy [5,6].

Nonetheless, amongst patients who undergo radical treatment for their primary tumour, between 22% and 38% will develop distant metastases [7]. Among patients with metastatic disease cancer, including BC, a distinct clinical subset exhibits limited spread—oligometastatic disease, an intermediate state between localised and widespread disease. In a large pan-cancer systemic review of 173 studies, the risk of progression and death is lower in oligometastatic disease compared to widespread metastatic disease, supporting its distinct classification [8]. However, oligometastatic bladder cancer (OMBC) has not been widely investigated and lacks a clear definition resulting in ambiguity in diagnosis and management. In BC-specific study, two-year cancer-specific survival (CSS) was as high as 53.3% versus 16.1% in those with polymetastatic disease, supporting the need for targeted treatment options [9]. However, survival outcomes within OMBC are heterogenous and influenced by metastatic site, burden, and treatment strategy. Most OMBC survival data are from highly selected cohorts treated in tertiary centres, limiting generalisability across healthcare systems with variable access to imaging and therapeutic options.

Despite increasing recognition, OMBC remains an ill-defined clinical entity, and its optimal management remains uncertain. The current literature varies in defining lesion number, size, anatomical distribution, and a limited integration of molecular or clinicopathological factors that may better predict prognosis and therapeutic response. This ambiguity hinders appropriate patient selection for treatment.

The increasing focus on precision oncology and biological stratification of metastatic states provides an opportunity to refine the definition and management of OMBC. The aim of this narrative review is to summarise the current understanding of OMBC, including its definitions, diagnostic approaches, and therapeutic pathways while highlighting key gaps that must be addressed through future prospective research.

## 2. Definitions of Oligometastatic Bladder Cancer

A major challenge in the landscape of OMBC is a lack of a universally accepted definition for the patient with oligometastatic cancer. Historically, Hellman and Weichesalbaum first described the oligometastatic state as an intermediate clinical state between localised and polymetastatic disease, which may be amenable to curative local therapy [10]. This was refined by Dingemans et al., who suggested a maximum of five metastases limited to three organs in their definition in non-small cell lung cancer [11]. However, this rigid, anatomical, definition does not factor in the unique nature of metastases depending on their location, genetic, epigenetic and immunologic phenotypes.

In urothelial carcinoma, definitions have largely been derived from retrospective series and expert consensus. The 2023 European Association of Urology (EAU)—European Society of Medical Oncology (ESMO)—European Society for Radiotherapy and Oncology (ESTRO) Delphi consensus study defined OMBC as a disease state with ≤3 metastatic sites, irrespective of organ involvement [12]. While no a priori consensus was reached regarding limits in lesion size, there was agreement that all sites should be resectable or amenable to stereotactic ablation. Pelvic lymph node disease was excluded, reflecting ongoing therapeutic uncertainties of their management following favourable response to chemotherapy [13]. Other prospective and retrospective series have adopted varying thresholds: Franzese et al. used ≤5 metastatic deposits in a stereotactic body radiation therapy (SBRT) trial, while Ogihara et al. and Augugliaro et al. restricted inclusion to ≤3 lesions [9,14,15,16,17]. Notably, Ogihara et al. applied a narrow definition of oligometastatic disease, restricting metastases to ≤3 lesions within a single organ, which limits generalizability to contemporary definitions [9]. The EAU-ESMO-ESTRO Delphi consensus study did not reach a consensus regarding lesion location [12]. Some authors have proposed excluding liver or brain metastases due to poorer outcomes, as evidence suggest that prognosis varies by metastatic site, with these sites having inferior OS compared to bone or lung disease [18,19,20]. In Australia, OMBC is not explicitly defined within national guidelines. Current Australian clinical practice largely follows international recommendations, including EAU guidance, with systemic therapy remaining the standard of care and MDT considered on a case-by-case basis within multidisciplinary teams.

Additionally, OMBC can be classified temporarily according to the timing of metastatic occurrence relative to the primary diagnosis. De novo synchronous OMBC refers to oligometastases within six months from the primary diagnosis; de novo metachronous oligorecurrence describes new oligometastases after treatment with curative intent of the primary lesion; and de novo metachronous oligoprogression refers to metastatic progression in a patient with metastatic disease controlled by systemic treatment or cytoreduction of other metastatic deposits.

**Figure 1:**
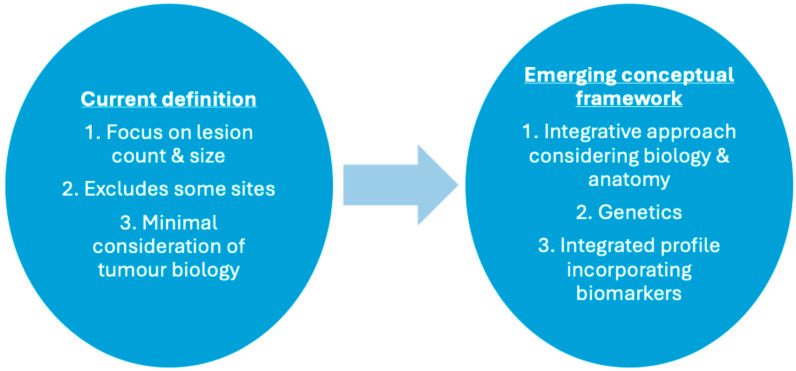
Comparison of traditional and emerging perspectives on OMBC.

However, definitions based solely on anatomical burden inadequately capture the biological heterogeneity of metastatic disease, with studies demonstrating significant inter- and intra-tumour heterogeneity in urothelial carcinoma, which included clonal evolution between primary and metastatic sites [21]. Molecular alterations, such as defects in DNA damage-repair (DDR) pathways, fibroblast growth factor receptor (FGFR) mutations, and epigenetic modifications, have been linked to differing metastatic behaviour and treatment sensitivities [22,23]. Additionally, the tumour microenvironment (TME), which includes immune infiltration and stromal responses, may influence metastatic potential and therapeutic response [24,25]. Recent systematic reviews emphasise the need for definitions that integrate molecular and clinicopathologic characteristics, alongside anatomical criteria [26,27]. Several international urological societies acknowledge OMBC, although few provide specific definitions or management pathways specific for OMBC (Table 1). Thus, a more nuanced, biologically informed classification system is necessary—one that factors lesion burden, location, and the molecular and genetic phenotype of tumours to guide personalised treatment (Figure 1).

Traditional definitions rely mainly on lesion count anatomical criteria, while contemporary research highlights the importance of integrating tumour biology, molecular and genetic profiling, and biomarkers to refine disease characterisation and optimise treatment selection for patients. While current definitions of OMBC remain predominately anatomical—based on metastatic lesion number, size, and distribution—emerging biological parameters are recognised as important modifiers rather than formal defining criteria. Molecular alterations, tumour microenvironment characteristics, and circulating biomarkers are not currently incorporated into definitions of OMBC. However, these markers may serve as surrogate indictors of disease kinetics and treatment responsiveness. Their inclusion in this review reflects growing interest in moving beyond pure anatomical classification towards a biologically informed framework that may refine patient selection not only in clinical trials, but also in specific treatment modalities.

## 3. Diagnostics

### 3.1. Imaging Modalities/Staging/Surveillance

Cross-sectional imaging remains pivotal in defining the extent of metastatic burden, and contrast-enhanced computed tomography (CT) of the chest, abdomen, and pelvis is the current standard of imaging [31]. However, CT has demonstrated variable sensitivity and specificity for detection of metastatic burden, particularly in small-volume lymph node or osseous disease. In their meta-analysis investigating the utility of CT, Yang et al. found a pooled per-patient sensitivity for metastases detection of 72.9%, per-lesion sensitivity of 77.1%, with a per-patient specificity of 94.8% and per-lesion specificity of 83.2% [32].

Recently, ^18^F-fluorodeoxyglucose-Positron Emission Tomography (FDG-PET)/CT and magnetic resonance imaging (MRI) have emerged as more sensitive tools for the detection of oligometastatic lesions compared with CT alone [33,34]. In their single-centre study of 133 patients, Shahait et al. found that FDG-PET/CT altered staging or treatment intent in 27.3% of cases, and that 24.7% of patients were downstaged from systemic to locoregional disease [35]. Accurate staging also aids in appropriate patient selection for neoadjuvant or metastasis-directed therapy (MDT) [34].

Despite these advantages, the use of FDG-PET/CT in primary staging of BC lacks sufficient evidence, with data from small retrospective studies and variable methodology employed. Furthermore, the sensitivity of FDG-PET/CT declines in the case of small lesions of <1 cm and in motion-affected organs such as the lung [36]. Meta-analyses evaluating FDG-PET/CT in urothelial carcinoma have reported pooled sensitivities ranging from 50 to 82% and specificity ranging from 89 to 91% for metastatic or nodal detection [37,38]. However, much of this data is limited by inter-protocol heterogeneity. Thus large, prospective studies are necessary to determine appropriate patient selection and imaging protocols. Several ongoing prospective studies (NCT05562791) aim to ascertain the role of novel tracers, including prostate-specific membrane antigen, in the systemic staging of BC [39].

Similarly, MRI has limitations given the lack of prospective evidence. One prospective pilot study examined 22 patients, reporting a sensitivity of 88% for lymph node detection using FDG-PET/MRI [40]. Nonetheless, MRI relies on size and morphological criteria to identify metastases, which can result in under-staging for micrometatstaic lymph nodes, as well as hepatic and pulmonary metastases [41]. Furthermore, a lack of whole-body standardisation limits utility in the detection of distant metastases. To augment standard imaging modalities, radiomics and artificial intelligence (AI) are being used for imaging analysis to enhance the diagnostic yield and detect micrometastatic disease, which may help in circumventing current limitations of modern imaging standards [42]. Radiomic analysis enables extraction of quantitative features from CT, PET, and MRI datasets, capturing tumour heterogeneity, metabolic activity, and spatial patterns that are not appreciable to the human eye [42]. Recent work in urothelial carcinoma has demonstrated the benefit of radiomics in predicting nodal involvement, metastatic potential, and treatment response [43]. Furthermore, AI-assisted imaging may aid in improving lesion characterisation, but evidence remains largely retrospective and heterogeneous, needing further validation [43].

Given the variable sensitivity of individual modalities, a complementary strategy is likely necessary in OMBC. CT remains appropriate for initial staging, while FDG-PET/CT offers advantages in detecting metabolically active nodal and osseous disease, particularly when lesion size approaches resolution limitations. Advanced imaging appears beneficial most for lymph nodes, bone, and equivocal visceral lesions, where management decisions hinge on accurate lesion identification.

### 3.2. Novel Biomarkers for Early Detection and Monitoring/Challenges of Biopsies and Molecular Testing

Tissue biopsy remains the gold standard in the confirmation of metastatic disease, but this can be challenging in OMBC due to the location of the tumour. Spatial heterogeneity among metastases remains a challenge, as different lesions may harbour distinct clonal or mutational profiles, and single-site biopsies may inadequately reflect the entire tumour landscape, leading to suboptimal therapeutic decision-making [44].

At present, no biomarkers reliably distinguish oligometastatic from polymetastatic disease states as discrete entities; rather, these states are considered part of a continuum of metastatic disease burden.

Liquid biopsy technologies are emerging as promising non-invasive tools to characterise disease kinetics and metastatic burden along this spectrum. Both circulating tumour cells (CTCs) and Circulating or Urinary Tumour DNA (ctDNA/utDNA) have shown prognostic value in oligometastatic disease more widely. In their prospective study, Sud et al. examined 43 patients with any solid malignancy and found that a lack of CTC clearance to ≤15/mL within 100 days of completing SBRT predicted disease progression [45]. Detection of ctDNA in the post-treatment setting in oligometastatic cancer has been shown to confer an increased risk of disease recurrence, lending promise to the use of ctDNA assays as a potential biomarker in future practice [46]. In the IMvigor010 and NIAGARA studies, ctDNA positivity identified patients at risk of poorer OS, supporting its role as a marker of minimal residual disease (MRD) [47,48]. In their recent update of the IMvigor010 trial, Powles et al. found that ctDNA positivity was associated with shorter OS in the control arm (HR 6.3, 95% CI: 4.3–9.3) and that Atezolizumab was associated with longer OS (HR 0.59, 95% CI: 0.42–0.83) [47]. Similarly, in NIAGARA, the presence of ctDNA positivity status pre-cystectomy was associated with a non-complete pathological response [48]. These findings are not unique to BC and have been validated in other malignancies—Chaudhuri et al. examined 40 patients with stage I-III lung cancer treated with curative intent, finding that in the 41% of patients with detectable ctDNA post-treatment, 100% experienced recurrence [49]. At present, no universally accepted quantitative ctDNA thresholds exist to define oligometastatic versus polymetastatic disease. Most studies interpret ctDNA qualitatively or by dynamic changes over time rather than by absolute copy number. While ctDNA is not yet incorporated into routine staging or OMBC definitions, its clinical utility is emerging in risk stratification and treatment escalation approaches. Ongoing work is necessary to validate whether ctDNA-guided intensification or de-escalation strategies can refine patient selection for systemic therapy and MDT approaches.

The superiority of utDNA over cytology and cystoscopy has been demonstrated in localised non-muscle-invasive bladder cancer [50]. While ctDNA/utDNA shows potential, their widespread adoption remains limited by low sensitivity in early stage, tumours with insufficient DNA sheading, and the inability to detect rare subclones. To overcomes these limitations, multi-analyte liquid biopsy assays are under investigation, combining ctDNA, methylation markers, and exosomal RNA to enhance predictive and prognostic value [51]. These novel approaches are in early clinical evaluation and validation to determine which patients are most at risk of polymetastatic progression. These biomarkers are not currently incorporated into formal definitions of OMBC, but their inclusion may provide further insight into treatment sensitivity and may complement anatomical criteria.

## 4. Treatment Strategies

### 4.1. Systemic Treatment Approaches

For patients with OMBC, platinum-based chemotherapy has historically represented the mainstay of first-line systemic therapy. Common regimes have included cistplatin/carboplatin and gemcitabine, followed by dose-dense methotrexate, vinblastine, doxorubicin, and cisplatin [ddMVAC] [52]. However, this landscape has evolved with the emergence of immune checkpoint inhibitors.

The role of combining systemic therapy and MDT remains relatively unexplored. In a small series, Muilwijk et al. found no OS benefit in those who had systemic treatment prior to metastectomy, although the small sample size limits interpretation [53].

Recent phase III trials have transformed the standard of care for metastatic BC. The EV-302 trial demonstrated a 31.5 month median (95% CI: 25.4–not reached) OS benefit (vs 16.1 months, 95% CI: 13.9–18.3 months) in those patients treated with Enfortumab Vedotin plus Pembrolizumab compared to Cisplatin/Gemcitabine alone [54]. Similarly, the Checkmate 901 trial demonstrated an OS and progression-free survival (PFS) benefit in those treated with Nivolumab plus Cisplatin and Gemcitabine compared to chemotherapy alone [4]. The recently reported NCT04570410 trial investigated Tislelizumab plus Gemcitabine/Cisplatin in 15 patients prior to cystectomy, achieving a 50% pathological complete response and 78% nodal clearance in patients with node-positive BC, supporting the feasibility of chemo-immunotherapy intensification in biologically selected, limited-burden disease [55].

Maintenance immunotherapy has become an integral component of treatment following upfront chemotherapy. The JAVELIN Bladder 100 trial reported that Avelumab maintenance improved OS (21.4 [95% CI: 18.9–26.1] months vs. 14.3 [95% CI: 12.9–17.9] months) compared to supportive care in patients with disease control after first-line chemotherapy [56].

There is ongoing research exploring systemic options through biomarker-driven and targeted approaches. Early trials of human epidermal growth factor 2 (HER-2)-directed therapies, such as Trastuzumab Deruxtecan and Disitamab Vedotin, have shown encouraging results in patients with HER2-positive disease in bladder cancer and other solid-tumours [57,58]. In advanced urothelial carcinoma, FGFR inhibitors such as Erdafitinib have demonstrated a durable response in patients whose tumours harbour FGFR 2/3 mutations [59]. These recent developments highlight a paradigm shift towards precision-based systemic therapy in advanced BC.

### 4.2. Consolidative Therapies

The role of consolidative surgery following induction chemotherapy has been well established in other malignancies yet remains relatively unexplored in OMBC [60]. Evidence from retrospective series suggest that cytoreductive surgery and consolidative radiotherapy may confer survival benefits in selected patients with limited metastatic burden [19,61].

In a retrospective series of 1381 patients with single-site metastases by Ji et al., cystectomy conferred a significant OS advantage compared with systemic therapy alone in those with bone or distant lymph node metastases, with a median survival of 15 and 21 months, respectively [19]. These findings were supported by Seisen et al. in their retrospective study of 603,298 men with metastatic BC [61]. Those who received either radical cystectomy or ≥50 Gy of radiotherapy (RT) had an OS improvement of approximately 5 months compared to those who received lower intensity therapy [61].

The rationale for consolidative local therapy lies in reducing residual tumour burden and the circulating tumour cell milieu, which may potentiate systemic therapy efficacy. This concept parallels findings in other malignancies, but high-quality prospective evidence remains lacking [62].

RT as a consolidative modality has shown encouraging evidence from retrospective series [63,64]. Abodudaram et al. evaluated 91 patients who remained progression-free following upfront systemic chemotherapy, of whom 51 received >50 Gy of RT to the bladder and residual metastases [63]. Consolidative RT improved both OS (HR 0.47, 95% CI: 0.25–0.86) and PFS (HR 0.49, 95% CI: 0.25–0.92) [63]. These findings are now being evaluated in a prospective multicentre Phase II randomised trial assessing the efficacy of consolidative RT in metastatic urothelial carcinoma [64].

Collectively, these data support further prospective exploration of multimodal, MDT in OMBC, particularly when systemic control has been achieved. Prospective trials integrating systemic and local consolidative therapies are warranted to define optimal treatment sequencing and patient selection.

### 4.3. Metastasis-Directed Therapy

The role of MDT, including metastasectomy and SBRT, in OMBC remains in an investigational phase. In a meta-analysis, Xing et al. found no OS benefit with metastasectomy compared to systemic therapy alone (HR 0.78, 95% CI: 0.56–1.08) [65]. However, anatomical site and disease burden appears to influence outcomes.

In their small retrospective series of 21 patients, Taguchi et al. observed that patients with liver metastases had a median survival of only 5 months, even among those with partial response to chemotherapy, whereas lung metastases were not associated with poorer survival in univariate analysis [66]. Conversely, Dong et al. in a population-based study of 1862 patients found that bone, brain, liver, and lung metastases were independent, negative prognostic factors for both OS and CSS [67].

Thus, the therapeutic benefit of metastasectomy likely depend on lesion number and site. Matsuguma et al. reported OS and PFS rates of 50% and 26%, respectively, following pulmonary metastasectomy in 32 patients with metastatic BC [68]. Similarly, Önder et al. found that resection of brain metastases in eight patients was associated with improved OS (HR 0.17, 95% CI 0.04–0.71), although the high incidence of widespread disease limits its overall benefit [69]. In patients unfit for surgery, thermal ablative techniques may provide durable local control in selected cases [70,71].

Cumulatively, current evidence for surgical MDT in OMBC is derived from small, retrospective cohorts or case series. While they may suggest potential benefit, these data require validation in prospective trials to identify patients most likely to derive benefit.

Historically, RT in metastatic BC has been reserved for palliation. However, contemporary data support its use as an ablative and potentially consolidative approach in the oligometastatic setting. Franzese et al. examined 61 patients across three centres treated using SBRT and reported PFS at 1 and 2 years of 47.9% and 38.1%, respectively, and a median OS of 25.6 months [14]. Similarly, in a systemic review of eight studies (n = 293), Angrisani et al. found a pooled 2-year OS of approximately 50.7% in patients treated with ablative doses (≥50 Gy) and low rates of grade ≥3 toxicity [72]. Recently, Svedman et al. demonstrated 82% local control and a median OS of 26.2 months among 39 patients treated with SBRT, with patients with solitary metastases achieving durable disease control [73]. Collectively, these findings highlight the potential of SBRT to achieve durable local control, prolong survival, and defer systemic therapy in well-selected OMBC patients [74].

Despite encouraging results, the evidence base remains predominantly retrospective, with small heterogenous cohorts and variable treatment schedules. Future, prospective randomised control trials are necessary to delineate optimal patient selection, treatment sequencing, and the integration of MDT with systemic therapy within a precision oncology framework.

### 4.4. Personalised Treatment

Personalised medicine is rapidly transforming the therapeutic landscape of OMBC. Comprehensive tumour genomic profiling has enabled the identification of actionable molecular alterations and therapeutic vulnerabilities. Erdafitinib has shown promise in patients whose tumours harbour FGFR 2/3 mutations, illustrating how molecular profiling can guide targeted therapy [75].

Immunotherapy personalisation has also advanced through biomarker-driven selection. The expression of programmed death-ligand 1 (PD-L1) and emerging genomic features such as DDR alterations have been associated with enhanced sensitivity to platinum-based chemotherapy and poly(adenosine-diphosphate-ribose) polymerase (PARP) inhibition [76].

In advanced urothelial carcinoma, ctDNA, tumour mutational burden, and mismatch repair deficiency are increasingly recognised as biomarkers of treatment response, particularly for immune checkpoint blockade [77].

The future of OMBC management lies in combining systemic therapy with MDT, guided by individual tumour biology. Computational and predictive models integrating genomic data, treatment history, and tumour evolution trajectories hold promise for optimising patient selection and treatment sequencing [78]. Multiple prospective trials are currently underway to evaluate biomarker-driven, multimodal approaches in OMBC, including OligoRARE (EORTC 1945) and NCT05259319, which aim to refine patient selection and individual treatment based on molecular and clinical features [79,80].

Long-term outcomes in OMBC remain poorly characterised. Available series suggest that recurrences most commonly occur at distant sites rather than only locally, supporting the concept that OMBC reflects systemic disease biology rather than only locoregional failure [14,72]. Durable disease control appears most likely in patients that achieve systemic control prior to MDT. Data on patient-reported outcomes and quality of life are notably scarce, despite their relevance when considering aggressive local therapies in metastatic settings. Future trials should incorporate patient-reported outcome measures, alongside survival endpoints to better inform shared decision-making and ensure an acceptable balance between oncologic benefit and treatment-related morbidity.

## 5. Conclusions

OMBC represents a biologically and clinically distinct state that bridges localised and widespread metastatic disease. Despite increasing recognition, OMBC remains poorly defined, with significant heterogeneity in diagnostic criteria, staging modalities, and therapeutic strategies. Evolving molecular diagnostics, advanced imaging, immunotherapy, and MDT are reshaping its management. Nonetheless, most available evidence derives from small retrospective series and expert consensus. Future progress depends on well-designed prospective and biomarker-driven trials to validate prognostic and predictive biomarkers and determine optimal integration and sequencing of systemic therapy with MDT. A biologically informed, precision-based framework is necessary to personalise therapy and improve outcomes for patients with OMBC.

## Figures and Tables

**Table 1:** Summary of international guideline recommendations regarding the recognition, diagnosis, and management of OMBC. Major urological associations (EAU, AUA, CUA) differ in recognising OMBC as a distinct clinical state, with limited consensus on lesion definition, imaging, and the role of local and metastasis-directed therapies, highlighting the need for standardised diagnostics and management frameworks.

Guidelines	Stance on OMBC	Imaging Modality Recommended	Recommended Management
European Association of Urology (EAU) [28]	Recognised as a distinct clinical entity. No strict consensus on number or location of lesions but proposed as ≤3 lesions amenable to radiotherapy or resection. Acknowledges limited information on OMBC.	No recommended modalities.	No specific recommendations. Acknowledges the utility of local therapy and MDT.
American Urological Association (AUA) [29]	Does not recognise OMBC as a distinct clinical state.	Recommends cross-sectional CT or MRI for staging.	Acknowledges that local therapy in metastatic therapy remains investigational. Emphasises systemic therapy as the cornerstone of management.
Canadian Urological Association (CUA) [30]	Recognised as a distinct clinical entity. No strict definition of lesion count mentioned.	Not discussed.	Encourages the use of multi-disciplinary approach in managing patients who have tumour regression following systemic therapy to determine utility of local therapy. Acknowledges the role of consolidative therapy and MDT. Emphasises need for prospective trials.

## Data Availability

No new data was generated.

## References

[1] Bray F., Laversanne M., Sung H., Ferlay J., Siegel R.L., Soerjomataram I., Jemal A. (2024). Global cancer statistics 2022: GLOBOCAN estimates of incidence and mortality worldwide for 36 cancers in 185 countries. CA Cancer J. Clin..

[2] Saginala K., Barsouk A., Aluru J.S., Rawla P., Padala S.A., Barsouk A. (2020). Epidemiology of Bladder Cancer. Med. Sci..

[3] von der Maase H., Hansen S.W., Roberts J.T., Dogliotti L., Oliver T., Moore M.J., Bodrogi I., Albers P., Knuth A., Lippert C.M. (2000). Gemcitabine and Cisplatin Versus Methotrexate, Vinblastine, Doxorubicin, and Cisplatin in Advanced or Metastatic Bladder Cancer: Results of a Large, Randomized, Multinational, Multicenter, Phase III Study. J. Clin. Oncol..

[4] van der Heijden M.S., Sonpavde G., Powles T., Necchi A., Burotto M., Schenker M., Sade J.P., Bamias A., Beuzeboc P., Bedke J. (2023). Nivolumab plus Gemcitabine-Cisplatin in Advanced Urothelial Carcinoma. N. Engl. J. Med..

[5] Powles T., Valderrama B.P., Gupta S., Bedke J., Kikuchi E., Hoffman-Censits J., Iyer G., Vulsteke C., Park S.H., Shin S.J. (2024). Enfortumab Vedotin and Pembrolizumab in Untreated Advanced Urothelial Cancer. N. Engl. J. Med..

[6] Powles T.B., Van der Heijden M.S., Loriot Y., Bedke J., Valderrama B.P., Iyer G., Kikuchi E., Hoffman-Censits J., Vulsteke C., Drakaki A. (2025). Enfortumab vedotin plus pembrolizumab in untreated locally advanced or metastatic urothelial carcinoma: 2.5-year median follow-up of the phase III EV-302/KEYNOTE-A39 trial. Ann. Oncol..

[7] Cagiannos I., Morash C. (2009). Surveillance strategies after definitive therapy of invasive bladder cancer. Can. Urol. Assoc. J..

[8] Petrelli F., Ghidini A., Ghidini M., Bukovec R., Trevisan F., Turati L., Indini A., Seghezzi S., Lonati V., Moleri G. (2021). Better survival of patients with oligo-compared with polymetastatic cancers: A systematic review and meta-analysis of 173 studies. F1000Research.

[9] Ogihara K., Kikuchi E., Watanabe K., Kufukihara R., Yanai Y., Takamatsu K., Matsumoto K., Hara S., Oyama M., Monma T. (2017). Can urologists introduce the concept of “oligometastasis” for metastatic bladder cancer after total cystectomy?. Oncotarget.

[10] Hellman S., Weichselbaum R.R. (1995). Oligometastases. J. Clin. Oncol..

[11] Dingemans A.C., Hendriks L.E.L., Berghmans T., Levy A., Hasan B., Faivre-Finn C., Giaj-Levra M., Giaj-Levra N., Girard N., Greillier L. (2019). Definition of Synchronous Oligometastatic Non-Small Cell Lung Cancer—A Consensus Report. J. Thorac. Oncol..

[12] Bamias A., Stenzl A., Brown S.L., Albiges L., Babjuk M., Birtle A., Briganti A., Burger M., Choudhury A., Colecchia M. (2023). Definition and Diagnosis of Oligometastatic Bladder Cancer: A Delphi Consensus Study Endorsed by the European Association of Urology, European Society for Radiotherapy and Oncology, and European Society of Medical Oncology Genitourinary Faculty. Eur. Urol..

[13] Necchi A., Mariani L., Lo Vullo S., Yu E.Y., Woods M.E., Wong Y.N., Harshman L.C., Alva A., Sternberg C.N., Bamias A. (2019). Lack of Effectiveness of Postchemotherapy Lymphadenectomy in Bladder Cancer Patients with Clinical Evidence of Metastatic Pelvic or Retroperitoneal Lymph Nodes Only: A Propensity Score-based Analysis. Eur. Urol. Focus..

[14] Franzese C., Francolini G., Nicosia L., Alongi F., Livi L., Scorsetti M. (2021). Stereotactic Body Radiation Therapy in the Management of Oligometastatic and Oligoprogressive Bladder Cancer and Other Urothelial Malignancies. Clin. Oncol. (R. Coll. Radiol.).

[15] Augugliaro M., Marvaso G., Ciardo D., Zerini D., Riva G., Rondi E., Vigorito S., Comi S., De Cobelli O., Orecchia R. (2019). Recurrent oligometastatic transitional cell bladder carcinoma: Is there room for radiotherapy?. Neoplasma.

[16] Francolini G., Desideri I., Detti B., Di Cataldo V., Masi L., Caramia G., Visani L., Terziani F., Muntoni C., Lo Russo M. (2019). Stereotactic radiotherapy in oligoprogressive and oligorecurrent urothelial cancer patients: A retrospective experience. Cancer Treat. Res. Commun..

[17] Miranda A.F., Howard J.M., McLaughlin M., Meng X., Clinton T., Sanli O., Garant A., Bagrodia A., Margulis V., Lotan Y. (2021). Metastasis-directed radiation therapy after radical cystectomy for bladder cancer. Urol. Oncol..

[18] Liu L., Yuan H., Wang Q., Li C. (2022). Effects of Different Organ Metastases on the Prognosis of Stage IV Urothelial Carcinoma of the Bladder. J. Oncol..

[19] Ji J., Lai C.H., Ni R., Wang M., Hu H., Bian X., Tian C., Wang C., Xu T., Hu H. (2025). Efficacy of cystectomy in single-site oligometastatic bladder cancer: A Surveillance, Epidemiology, and End Results (SEER) study of 1381 patients. Transl. Androl. Urol..

[20] Di Bello F., de Angelis M., Siech C., Jannello L.M.I., Penaranda N.R., Tian Z., Goyal J.A., Ruvolo C., Califano G., La Rocca R. (2024). Survival of Metastatic Urothelial Carcinoma of Urinary Bladder According to Number and Location of Visceral Metastases. Clin. Genitourin. Cancer.

[21] Kang H.W., Kim W.J., Yun S.J. (2020). The therapeutic and prognostic implications of molecular biomarkers in urothelial carcinoma. Transl. Cancer Res..

[22] Al-Obaidy K.I., Cheng L. (2021). Fibroblast growth factor receptor (FGFR) gene: Pathogenesis and treatment implications in urothelial carcinoma of the bladder. J. Clin. Pathol..

[23] Teo M.Y., Mota J.M., Whiting K.A., Li H.A., Funt S.A., Lee C.-H., Solit D.B., Al-Ahmadie H., Milowsky M.I., Balar A.V. (2020). Fibroblast Growth Factor Receptor 3 Alteration Status is Associated with Differential Sensitivity to Platinum-based Chemotherapy in Locally Advanced and Metastatic Urothelial Carcinoma. Eur. Urol..

[24] Hatogai K., Sweis R.F. (2020). The Tumor Microenvironment of Bladder Cancer. Adv. Exp. Med. Biol..

[25] Ahmed B.H., Zdyrski C., Cheville J., Rancilio N., Abel A.M., Wierson W.A., McGill J.L., Mochel J.P., Allenspach K. (2023). Role of the tumour microenvironment in bladder cancer pathogenesis and value of the reverse translational approach: A tale of two species. Clin. Transl. Discov..

[26] Bamias A., Stenzl A., Zagouri F., Andrikopoulou A., Hoskin P. (2023). Defining Oligometastatic Bladder Cancer: A Systematic Review. Eur. Urol. Open Sci..

[27] Guckenberger M., Lievens Y., Bouma A.B., Collette L., Dekker A., deSouza N.M., Dingemans A.C., Fournier B., Hurkmans C., Lecouvet F.E. (2020). Characterisation and classification of oligometastatic disease: A European Society for Radiotherapy and Oncology and European Organisation for Research and Treatment of Cancer consensus recommendation. Lancet Oncol..

[28] van der Heijden A.G., Bruins H.M., Carrion A., Cathomas R., Compérat E.M., Dimitropoulos K., Efstathiou J.A., Fietkau R., Lorch A., Mariappan P. (2025). EAU Guidelines on Muscle-Invasive and Metastatic Bladder Cancer.

[29] Chang S.S., Bochner B.H., Chou R., Dreicer R., Kamat A.M., Lerner S.P., Lotan Y., Meeks J.J., Michalski J.M., Morgan T.M. (2017). Treatment of Non-Metastatic Muscle-Invasive Bladder Cancer: AUA/ASCO/ASTRO/SUO Guideline. J. Urol..

[30] Stecca C.E., Chowdhury D., Blais N., Alimohamed N., Wood L., Canil C.M., Eigl B., Kulkarni G.S., Black P.C., Kassouf W. (2024). 2024 CUA-GUMOC Expert Report: Management of unresectable locally advanced and metastatic urothelial carcinoma. Can. Urol. Assoc. J..

[31] Alfred Witjes J., Lebret T., Compérat E.M., Cowan N.C., De Santis M., Bruins H.M., Hernández V., Espinós E.L., Dunn J., Rouanne M. (2017). Updated 2016 EAU Guidelines on Muscle-invasive and Metastatic Bladder Cancer. Eur. Urol..

[32] Yang H.L., Liu T., Wang X.M., Xu Y., Deng S.M. (2011). Diagnosis of bone metastases: A meta-analysis comparing ^18^FDG PET, CT, MRI and bone scintigraphy. Eur. Radiol..

[33] Apolo A.B., Riches J., Schoder H., Akin O., Trout A., Milowsky M.I., Bajorin D.F. (2010). Clinical value of fluorine-18 2-fluoro-2-deoxy-D-glucose positron emission tomography/computed tomography in bladder cancer. J. Clin. Oncol..

[34] Crozier J., Papa N., Perera M., Ngo B., Bolton D., Sengupta S., Lawrentschuk N. (2019). Comparative sensitivity and specificity of imaging modalities in staging bladder cancer prior to radical cystectomy: A systematic review and meta-analysis. World J. Urol..

[35] Shahait M., Abu-Hijlih R., Farkouh A., Obeidat S., Salah S., Abdlkadir A.S., Al-Ibraheem A. (2023). Fluorodeoxyglucose positron emission tomography (18F-FDG PET)-computed tomography (CT) in the initial staging of bladder cancer: A single institution experience. J. Egypt. Natl. Cancer Inst..

[36] Gonzalez-Del-Alba A., Conde-Moreno A.J., Garcia Vicente A.M., Gonzalez-Peramato P., Linares-Espinos E., Climent M.A., The Sogug Multidisciplinary Working Group (2022). Management of Patients with Metastatic Bladder Cancer in the Real-World Setting from the Multidisciplinary Team: Current Opinion of the SOGUG Multidisciplinary Working Group. Cancers.

[37] Lu Y.Y., Chen J.H., Liang J.A., Wang H.Y., Lin C.C., Lin W.Y., Kao C.H. (2012). Clinical value of FDG PET or PET/CT in urinary bladder cancer: A systemic review and meta-analysis. Eur. J. Radiol..

[38] Abdlkadir A.S., Al-Adhami D., Allouzi S., Badarneh M., Shahait M., Abdulrahman M., Ruzzeh S., Moghrabi S., Al-Ibraheem A. (2025). Diagnostic efficacy of [^18^F]FDG PET/CT and [^18^F]FDG PET/MRI in preoperative staging of locoregional urinary bladder cancer: A systematic review and Meta-Analysis. Discov. Oncol..

[39] (2022). Pilot Study of 68Gallium PSMA-PET/CT in Patients with Metastatic Urothelial Carcinoma or Melanoma. https://clinicaltrials.gov/study/NCT05562791.

[40] Rosenkrantz A.B., Friedman K.P., Ponzo F., Raad R.A., Jackson K., Huang W.C., Balar A.V. (2017). Prospective Pilot Study to Evaluate the Incremental Value of PET Information in Patients with Bladder Cancer Undergoing 18F-FDG Simultaneous PET/MRI. Clin. Nucl. Med..

[41] Woo S., Suh C.H., Kim S.Y., Cho J.Y., Kim S.H. (2018). The Diagnostic Performance of MRI for Detection of Lymph Node Metastasis in Bladder and Prostate Cancer: An Updated Systematic Review and Diagnostic Meta-Analysis. Am. J. Roentgenol..

[42] Gillies R.J., Kinahan P.E., Hricak H. (2016). Radiomics: Images Are More than Pictures, They Are Data. Radiology.

[43] Sun R., Zhang M., Yang L., Yang S., Li N., Huang Y., Song H., Wang B., Huang C., Hou F. (2024). Preoperative CT-based deep learning radiomics model to predict lymph node metastasis and patient prognosis in bladder cancer: A two-center study. Insights Imaging.

[44] Gerlinger M., Rowan A.J., Horswell S., Math M., Larkin J., Endesfelder D., Gronroos E., Martinez P., Matthews N., Stewart A. (2012). Intratumor heterogeneity and branched evolution revealed by multiregion sequencing. N. Engl. J. Med..

[45] Sud S., Poellmann M.J., Hall J., Tan X., Bu J., Myung J.H., Wang A.Z., Hong S., Casey D.L. (2023). Prospective Characterization of Circulating Tumor Cell Kinetics in Patients with Oligometastatic Disease Receiving Definitive Intent Radiation Therapy. JCO Precis. Oncol..

[46] Routman D.M., Chera B.S., Gupta G.P. (2020). Circulating Tumor DNA Biomarkers for Early Detection of Oligometastasis. Cancer J..

[47] Powles T., Assaf Z.J., Degaonkar V., Grivas P., Hussain M., Oudard S., Gschwend J.E., Albers P., Castellano D., Nishiyama H. (2024). Updated Overall Survival by Circulating Tumor DNA Status from the Phase 3 IMvigor010 Trial: Adjuvant Atezolizumab Versus Observation in Muscle-invasive Urothelial Carcinoma. Eur. Urol..

[48] Powles T., Van Der Heijden M.S., Wang Y., Catto J.W.F., Meeks J.J., Al-Ahmadie H., Nishiyama H., Moeini Mortazavi A., Vu T.Q., Antonuzzo L. (2025). Circulating tumor DNA (ctDNA) in patients with muscle-invasive bladder cancer (MIBC) who received perioperative durvalumab (D) in NIAGARA. J. Clin. Oncol..

[49] Chaudhuri A.A., Chabon J.J., Lovejoy A.F., Newman A.M., Stehr H., Azad T.D., Khodadoust M.S., Esfahani M.S., Liu C.L., Zhou L. (2017). Early Detection of Molecular Residual Disease in Localized Lung Cancer by Circulating Tumor DNA Profiling. Cancer Discov..

[50] Dudley J.C., Schroers-Martin J., Lazzareschi D.V., Shi W.Y., Chen S.B., Esfahani M.S., Trivedi D., Chabon J.J., Chaudhuri A.A., Stehr H. (2019). Detection and Surveillance of Bladder Cancer Using Urine Tumor DNA. Cancer Discov..

[51] Wan J.C.M., Massie C., Garcia-Corbacho J., Mouliere F., Brenton J.D., Caldas C., Pacey S., Baird R., Rosenfeld N. (2017). Liquid biopsies come of age: Towards implementation of circulating tumour DNA. Nat. Rev. Cancer.

[52] Flaig T.W., Spiess P.E., Agarwal N., Bangs R., Boorjian S.A., Buyyounouski M.K., Chang S., Downs T.M., Efstathiou J.A., Friedlander T. (2020). Bladder Cancer, Version 3.2020, NCCN Clinical Practice Guidelines in Oncology. J. Natl. Compr. Cancer Netw..

[53] Muilwijk T., Akand M., Van der Aa F., Dumez H., De Meerleer G., Van Raemdonck D., De Leyn P., Van Poppel H., Albersen M., Joniau S. (2020). Metastasectomy of oligometastatic urothelial cancer: A single-center experience. Transl. Androl. Urol..

[54] Powles T.B., Perez Valderrama B., Gupta S., Bedke J., Kikuchi E., Hoffman-Censits J., Iyer G., Vulsteke C., Park S.H., Shin S.J. (2023). LBA6 EV-302/KEYNOTE-A39: Open-label, randomized phase III study of enfortumab vedotin in combination with pembrolizumab (EV+P) vs. chemotherapy (Chemo) in previously untreated locally advanced metastatic urothelial carcinoma (la/mUC). Ann. Oncol..

[55] Yang X., Zhuang J., Cao Q., Cai L., Wu Q., Lu Q. (2024). Tislelizumab in combination with gemcitabine plus cisplatin as neoadjuvant therapy for lymph node-positive bladder cancer: Results of a prospective study. J. Clin. Oncol..

[56] Powles T., Park S.H., Voog E., Caserta C., Valderrama B.P., Gurney H., Kalofonos H., Radulovic S., Demey W., Ullen A. (2020). Avelumab Maintenance Therapy for Advanced or Metastatic Urothelial Carcinoma. N. Engl. J. Med..

[57] Meric-Bernstam F., Makker V., Oaknin A., Oh D.Y., Banerjee S., Gonzalez-Martin A., Jung K.H., Lugowska I., Manso L., Manzano A. (2024). Efficacy and Safety of Trastuzumab Deruxtecan in Patients with HER2-Expressing Solid Tumors: Primary Results from the DESTINY-PanTumor02 Phase II Trial. J. Clin. Oncol..

[58] Zhou Y.X., Wang J.L., Mu X.L., Zhu Y.J., Chen Y., Liu J.Y. (2023). Efficacy and safety analysis of a HER2-targeting antibody-drug conjugate combined with immune checkpoint inhibitors in solid tumors: A real-world study. Aging.

[59] Loriot Y., Matsubara N., Park Se H., Huddart Robert A., Burgess Earle F., Houede N., Banek S., Guadalupi V., Ku Ja H., Valderrama Begoña P. (2023). Erdafitinib or Chemotherapy in Advanced or Metastatic Urothelial Carcinoma. N. Engl. J. Med..

[60] Morris V.K., Kennedy E.B., Baxter N.N., Benson A.B., Cercek A., Cho M., Ciombor K.K., Cremolini C., Davis A., Deming D.A. (2023). Treatment of Metastatic Colorectal Cancer: ASCO Guideline. J. Clin. Oncol..

[61] Seisen T., Sun M., Leow J.J., Preston M.A., Cole A.P., Gelpi-Hammerschmidt F., Hanna N., Meyer C.P., Kibel A.S., Lipsitz S.R. (2016). Efficacy of High-Intensity Local Treatment for Metastatic Urothelial Carcinoma of the Bladder: A Propensity Score-Weighted Analysis from the National Cancer Data Base. J. Clin. Oncol..

[62] Hiratsuka S., Watanabe A., Aburatani H., Maru Y. (2006). Tumour-mediated upregulation of chemoattractants and recruitment of myeloid cells predetermines lung metastasis. Nat. Cell Biol..

[63] Aboudaram A., Chaltiel L., Pouessel D., Graff-Cailleaud P., Benziane-Ouaritini N., Sargos P., Schick U., Créhange G., Cohen-Jonathan Moyal E., Chevreau C. (2023). Role of consolidative radiotherapy for metastatic urothelial bladder cancer patients without progression and with no more than five residual metastatic lesions following first line systemic therapy: A retrospective analysis. Radiother. Oncol..

[64] Khalifa J., Pouessel D., Roumiguie M., Sargos P., Loos G., Schick U., Salem N., Mesgouez-Nebout N., Loriot Y., Hennequin C. (2021). Consolidative radiotherapy for metastatic urothelial bladder cancer patients without progression and with no more than three residual metastatic lesions following first line systemic therapy: A prospective randomized comparative phase II trial (BLAD RAD01/GETUG-AFU V07). J. Clin. Oncol..

[65] Xing Q., Ji C., Wang Y., Wang X., Zhu Z. (2020). Metastasectomy could not improve the survival of metastatic urothelial carcinoma: Evidence from a meta-analysis. Transl. Cancer Res..

[66] Taguchi S., Nakagawa T., Matsumoto A., Nagase Y., Kawai T., Tanaka Y., Yoshida K., Yamamoto S., Enomoto Y., Nose Y. (2015). Pretreatment neutrophil-to-lymphocyte ratio as an independent predictor of survival in patients with metastatic urothelial carcinoma: A multi-institutional study. Int. J. Urol..

[67] Dong F., Shen Y., Gao F., Xu T., Wang X., Zhang X., Zhong S., Zhang M., Chen S., Shen Z. (2017). Prognostic value of site-specific metastases and therapeutic roles of surgery for patients with metastatic bladder cancer: A population-based study. Cancer Manag. Res..

[68] Matsuguma H., Yoshino I., Ito H., Goya T., Matsui Y., Nakajima J., Ikeda N., Okumura S., Shiono S., Nomori H. (2011). Is There a Role for Pulmonary Metastasectomy with a Curative Intent in Patients with Metastatic Urinary Transitional Cell Carcinoma?. Ann. Thorac. Surg..

[69] Onder T., Karacin C., Kekilli E., Goksel F., Sertesen E., Onur I.D., Ates O., Yildiz F., Arslan U.Y. (2024). Predicting survival after brain metastases in patients with bladder cancer. J. Clin. Neurosci..

[70] Ahmed M., Solbiati L., Brace C.L., Breen D.J., Callstrom M.R., Charboneau J.W., Chen M.-H., Choi B.I., de Baère T., Dodd G.D. (2014). Image-guided tumor ablation: Standardization of terminology and reporting criteria—A 10-year update: Supplement to the consensus document. J. Vasc. Interv. Radiol..

[71] Erwin X., Ambrose J.H. (2023). Thermal ablation of metastatic disease to the musculoskeletal system. J. Cancer Metastasis Treat..

[72] Angrisani A., Bosetti D.G., Vogl U.M., Castronovo F.M., Zilli T. (2024). Oligometastatic Urothelial Cancer and Stereotactic Body Radiotherapy: A Systematic Review and an Updated Insight of Current Evidence and Future Directions. Cancers.

[73] Svedman F.C., Holmsten K., Jawdat F., Hailom W., Alm D., Grozman V., Ullén A. (2024). Stereotactic body radiation therapy is beneficial for a subgroup of patients with urothelial cancer and solitary metastatic disease: A single institution real-world experience. Radiat. Oncol..

[74] Carriere P., Alhalabi O., Gao J., Shah A.Y., Campbell M.T., Mayo L.L., Mohamad O., Hoffman K.E., Mok H., Lozano C. (2025). Metastasis-directed radiotherapy in oligometastatic bladder and upper tract cancer: A single institution retrospective experience. J. Clin. Oncol..

[75] Loriot Y., Necchi A., Park S.H., Garcia-Donas J., Huddart R., Burgess E., Fleming M., Rezazadeh A., Mellado B., Varlamov S. (2019). Erdafitinib in Locally Advanced or Metastatic Urothelial Carcinoma. N. Engl. J. Med..

[76] Teo M.Y., Seier K., Ostrovnaya I., Regazzi A.M., Kania B.E., Moran M.M., Cipolla C.K., Bluth M.J., Chaim J., Al-Ahmadie H. (2018). Alterations in DNA Damage Response and Repair Genes as Potential Marker of Clinical Benefit from PD-1/PD-L1 Blockade in Advanced Urothelial Cancers. J. Clin. Oncol..

[77] Powles T., Duran I., van der Heijden M.S., Loriot Y., Vogelzang N.J., De Giorgi U., Oudard S., Retz M.M., Castellano D., Bamias A. (2018). Atezolizumab versus chemotherapy in patients with platinum-treated locally advanced or metastatic urothelial carcinoma (IMvigor211): A multicentre, open-label, phase 3 randomised controlled trial. Lancet.

[78] Doroshow J.H., Kummar S. (2014). Translational research in oncology—10 years of progress and future prospects. Nat. Rev. Clin. Oncol..

[79] (2020). Stereotactic Body Radiotherapy in Addition to Standard of Care Treatment in Patients with Rare Oligometastatic Cancers (OligoRARE): A Randomized, Phase 3, Open-Label Trial. https://clinicaltrials.gov/study/NCT04498767.

[80] Roussot N., Fumet J.D., Limagne E., Thibaudin M., Hervieu A., Hennequin A., Zanetta S., Dalens L., Fourrier T., Galland L. (2023). A phase I study of the combination of atezolizumab, tiragolumab, and stereotactic body radiation therapy in patients with metastatic multiorgan cancer. BMC Cancer.

